# Age-Dependent Association Between Elevated Homocysteine and Cognitive Impairment in a Post-stroke Population: A Prospective Study

**DOI:** 10.3389/fnut.2021.691837

**Published:** 2021-06-29

**Authors:** Shengnan Zhou, Jiahao Chen, Lin Cheng, Kaili Fan, Minjie Xu, Wenwei Ren, Yunbin Chen, Dandan Geng, Haoran Cheng, Xiaoqian Luan, Jiaying Song, Gangqiang Lin, Guiqian Huang, Jincai He

**Affiliations:** ^1^Department of Neurology, The First Affiliated Hospital of Wenzhou Medical University, Wenzhou, China; ^2^School of Mental Health, Wenzhou Medical University, Wenzhou, China

**Keywords:** homocysteine, age, ischemic stroke, cognitive impairment, Mini-Mental State Exam

## Abstract

**Background and Purpose:** The results regarding the independent association between homocysteine (Hcy) levels and post-stroke cognitive impairment (PSCI) were inconsistent. The effect of age on this association has yet to be explored. This study aims to determine the relationship between Hcy levels, age, and cognitive impairment in a post-stroke population.

**Methods:** A total of 592 patients with acute ischemic stroke (AIS) completed follow-up. Serum Hcy levels were measured enzymatically by spectrophotometry within 24 h of admission. Cognitive function was evaluated by the Mini-Mental State Examination (MMSE) 1 month after stroke, and the scores ≤ 24 were considered as cognitive impairment. Our study was dichotomized into two groups by a cut-off of 65 years. Multivariate logistic regression models were used to determine the association between baseline Hcy levels and cognitive impairment.

**Results:** According to the MMSE score, 317 (53.5%) patients had cognitive impairment. Patients with higher levels of Hcy were more prone to have cognitive impairment 1 month after stroke than patients with lower levels of Hcy (*p* < 0.001). The optimal cut-off points of Hcy level (μmol/L) were (T1) ≤ 8, (T2) 8–12, and (T3) ≥ 12. After adjusting for confounding factors, the multivariate regression analysis showed that the third Hcy tertile was independently associated with cognitive impairment [odds ratio (OR) = 2.057, 95% confidence interval (CI) = 1.133–3.735, *p* = 0.018). A stronger association [T2 (OR = 2.266, 95% CI = 1.042–4.926, *p* = 0.039); T3 (OR =3.583, 95% CI = 1.456–8.818, *p* = 0.005)] was found in the younger group. However, the independent association was not confirmed in the older group.

**Conclusions:** Elevated Hcy levels in the acute phase of ischemic stroke were independently associated with cognitive impairment in a post-stroke population. Furthermore, the association was age-dependent and more meaningful in a younger population aged below 65. So, Hcy levels in patients with stroke should be well-monitored, especially in younger patients.

## Introduction

Currently, the prevalence of post-stroke cognitive impairment (PSCI) ranges from 20 to 80% among different countries, races, and diagnostic criteria (1), which is associated with functional outcomes and survival of stroke (2, 3) resulting in a tremendous clinical and economic burden on individuals and society. Moreover, cognitive impairment tends to progressively worsen following stroke (4), with 20–30% of the patients developing dementia (5). Hence, international guidelines recommend cognitive assessment as a routine neurological examination for all stroke survivors (6). Older age, poor educational status, female sex, and genetic factors are known conventional unamenable risk factors for PSCI (7). Hence, it is imperative to identify modifiable risk factors to guide medical treatments and reduce the prevalence of post-stroke dementia (PSD). Concentrations of homocysteine (Hcy) represent a modifiable risk factor that can be prevented and treated by vitamin supplementation. Therefore, understanding the prognostic impact of Hcy levels for PSCI is clinically relevant.

A growing body of evidence suggests that high Hcy levels may contribute to the pathogenesis of PSCI *via* oxidative stress (8), vascular inflammation (8), endothelial dysfunction (9, 10), and accelerate amyloid and tau protein accumulation (11). However, clinical studies investigating the association between Hcy levels and cognitive impairment in patients with stroke have yielded inconsistent results (12–16). The reasons for the contradictory results of previous studies remain equivocal. Age is one possible explanation for this discrepancy. Notably, Hcy concentrations were reported to increase with age (17, 18). Numerous studies have shown that age not only affects the relationship between Hcy and stroke (19–21) but also the relationship between Hcy and cognitive decline (22). For example, a nationwide study based on 12,683 patients with stroke suggested that the association between Hcy and stroke was strongest in younger individuals and declined linearly with increasing age (20). To the best of our knowledge, the effect of age on the association between Hcy and PSCI has yet to be explored. A traditional threshold of age at which people can be assumed to be “old,” the age of 65 years, was linked with Alzheimer's disease (AD) (23) and PSD (4). To moderate the confounding effect of age, our study was dichotomized into two groups by a cut-off of 65 years.

Therefore, this study was designed to determine the relationship between Hcy levels, age, and cognitive impairment in a post-stroke population. In other words, we aimed to identify the association between Hcy levels and cognitive impairment in a post-stroke population and investigate the effect of age on the association.

## Method

### Study Design and Participants

From October 2013 to November 2019, patients with acute ischemic stroke (AIS) were consecutively recruited within 7 days of onset at the First Affiliated Hospital of Wenzhou Medical University. Eligible participants with AIS aged between 18 and 80 years were diagnosed using CT or MRI within 72 h of admission and were willing to support our work by completing the follow-up plans and cognitive assessments.

All the participants completed the detection of related indicators within 24 h of admissions, such as folate, vitamin B12, and Hcy. The Hcy levels were further divided into tertiles according to the number of patients and the distribution of the Hcy value, which makes the highest differences in this study. Furthermore, the Hcy tertiles were used to observe whether any enhanced performance could be quantified while maintaining the statistical efficacy in each category.

The exclusion criteria were as follows: (1) pre-existing transient ischemic attack or hemorrhagic stroke history, (2) intravenous thrombolytic therapy or interventional treatment received by the patient, (3) primary major cognitive impairment even dementia, (4) other central nervous system diseases, such as Parkinson disease and hydrocephalus, (5) serious mental illness, including pre-stroke depression and schizophrenia, (6) severe critical organ failure, especially severe kidney disease, (7) out of competence to complete cognitive assessments, such as coma, severe aphasia, dysarthria or hearing impairment, and (8) folate or vitamin B therapy within 2 weeks of admission or medication therapy that would affect Hcy levels. Ultimately, a total of 592 patients completed the follow-up and were included in this study.

This prospective cohort study was approved by the Ethics Committee of the First Affiliated Hospital of Wenzhou Medical University and was conducted in accordance with the ethical guidelines of the 1975 Declaration of Helsinki. All participants or their guardians understood our study well and signed written informed consent. For participants with less education or cognitive impairment, verbal informed consent was provided to them together with their guardians. Finally, with the full understanding and agreement of guardians, those participants who could not write signed a written informed consent using their thumbprints. Meanwhile, their guardians signed their names beside the thumbprints.

### Data Collection

Baseline information was acquired through face-to-face interviews combined with electronic medical records and primary nursing records. A standardized questionnaire was designed to collect information on the demographic characteristics of trained neurological physicians. Demographic data included age, sex, body mass index (BMI), and years of education. Vascular risk factors included currently smoking, currently drinking, hypertension, diabetes mellitus, coronary artery disease (CAD), and atrial fibrillation (AF). Notably, blood pressure (BP) was recorded within 24 h after admission as systolic blood pressure (SBP) and diastolic blood pressure (DBP). Laboratory tests included Hcy, folate, vitamin B12, and estimated glomerular filtration rate (eGFR). The serum levels of Hcy, folate, and vitamin B12 were measured using an automatic biochemical analyzer (Beckman Olympus AU2700, USA) at the laboratory of our hospital. The concentrations were analyzed enzymatically using spectrophotometry with commercial reagents.

Other clinical data we collected included stroke severity, TOAST mechanism, and clinical depressive symptoms. However, this study lacked the Mini-Mental State Examination (MMSE) scores before the onset of stroke. The patients included in this study already had a stroke before admission to the Department of Neurology, so we were unable to collect the MMSE scores before the stroke. Although we excluded patients with mild cognitive impairment or dementia from this study, there were still potential confounders of pre-stroke cognitive impairment.

Stroke severity was assessed on admission by experienced neurologists using the National Institutes of Health Stroke Scale (NIHSS) (24). All the patients were investigated to clarify the stroke subtype according to the TOAST criteria (25). The severity of depression of patients was evaluated by Hamilton Depression Scale (HAMD-17) (26) within 7 days of onset, which was recorded as the HAMD score. According to the Diagnostic and Statistical Manual of Mental Disorders, Fifth, a HAMD score ≥ 7 combined with clinical manifestations indicated depressive symptoms.

A radiologist who was blinded to the clinical results performed the cranial CT or MRI on patients within 72 h after admission.

### Outcome Assessment and Follow-Up

Experienced neurological physicians, blinded to the baseline characteristics of the patients, evaluated the cognitive function of the patients using the Chinese version of the MMSE (27) at 1 month after stroke. The MMSE has been translated into Chinese and was validated for reliability and validity as a screening tool for cognitive impairment in the Chinese stroke population (28). The MMSE scores range from 0 to 30, with higher scores indicating better performance. Considering the low education level of the patients, the MMSE scores ≤ 24 points indicated cognitive impairment (29–32).

### Statistical Analyses

Categorical variables were shown as proportions, and group differences were analyzed using the chi-square test. According to the distribution of continuous data, mean ± SD was used to describe a normal distribution, and Student's *t*-test was used to assess the group differences. For skewed distribution, median (interquartile range, IQR) and Mann–Whitney *U*-test were implemented. According to the Hcy tertiles, all the patients were divided into three groups and the comparisons among the three tertiles were performed using the Kruskal–Wallis-test, one-way ANOVA, Pearson's chi-square-test, or Fisher's exact-test. Variables reflecting significant group differences (*p* < 0.05) were considered as confounding factors and were included in the univariate and multivariate logistic regression analyses. Model 1 included age, sex, and years of education. Model 2 was adjusted for age, sex, years of education, currently smoking, currently drinking, hypertension, AF, folate, eGFR, NIHSS score, TOAST mechanism, and HAMD score. Model 3 was adjusted for sex and years of education. Model 4 was adjusted for sex, years of education, currently smoking, currently drinking, hypertension, AF, folate, eGFR, NIHSS score, TOAST mechanism, and HAMD score. Two-tailed *p*-values < 0.05 were considered statistically significant, which were computed using IBM SPSS Statistics software Version 26 for Windows.

## Result

### Baseline Characteristics of Patients With AIS Grouped by Cognitive Impairment According to MMSE

During the research period, a total of 1,112 patients with AIS were enrolled in this prospective study, of which 705 patients were eligible for the study. Ultimately, 592 patients completed the 1-month follow-up and were included in this study. A total of 222 (37.5%) patients were female, and the median age of the enrolled patients was 64 years (range 30–80 years), and the median Hcy concentration was 10.0 μmol/L (range 3.1–38.0 μmol/L). In addition, 317 (53.5%) patients had cognitive impairment, which is similar to a number of previous research (1, 33).

Table 1 shows the baseline demographic, clinical, and laboratory characteristics of patients with and without cognitive impairment. Compared to patients without cognitive impairment, patients with cognitive impairment were more likely to be older and female. Also, they tended to have fewer years of education and higher NIHSS scores. Meanwhile, fewer cigarette smokers and alcohol drinkers were found in patients with cognitive impairment, but patients with a history of hypertension and AF were more likely to undergo cognitive impairment. Regarding laboratory parameters, patients with cognitive impairment had higher serum Hcy concentration, but lower serum folate concentrations and lower eGFR. Among patients with and without cognitive impairment, the median (IQR) values of Hcy were 10.9 μmol/L (8.0–13.0 μmol/L) and 9.5 μmol/L (4.0–12.0 μmol/L), respectively, and showed a significant difference (*p* = 0.001). Furthermore, patients with cognitive impairment had higher HAMD scores at baseline, reflecting a worse emotional status of these patients compared to those without cognitive impairment.

**Table 1:** Baseline characteristics of patients with AIS grouped by cognitive impairment according to MMSE.

**Variables**	**Total**	**MMSE > 24**	**MMSE ≤ 24**	***P*-value**
	**(*n* = 592)**	**(*n* = 275)**	**(*n* = 317)**	
Age, year (median, IQR)	64.0 (57.0–70.0)	61.0 (51.0–67.0)	67.0 (60.0–73.0)	<0.001
Female, *n* (%)	222 (37.5%)	60 (21.8%)	162 (51.1%)	<0.001
BMI (median, IQR)	23.8 (22.0–26.1)	24.0 (22.1–26.3)	23.4 (21.6–26.0)	0.079
Education, year (median, IQR)	4.0 (0.0–6.0)	6.0 (4.0–9.0)	0.0 (0.0–5.0)	<0.001
Currently smoking, *n* (%)	274 (46.8%)	158 (58.3%)	116 (36.9%)	0.002
Currently drinking, *n* (%)	206 (36.1%)	111 (41.9%)	95 (31.0%)	0.007
Hypertension, *n* (%)	421 (71.1%)	184 (66.9%)	237 (74.8%)	0.035
Hyperlipidemia, *n* (%)	49 (8.3%)	27 (9.8%)	21 (6.7%)	0.170
Diabetes, *n* (%)	138 (23.5%)	63 (22.9%)	75 (24.0%)	0.764
CAD, *n* (%)	38 (6.5%)	18 (6.6%)	20 (6.4%)	0.902
AF, *n* (%)	47 (7.9%)	15 (5.5%)	32 (10.1%)	0.037
SBP, mmHg (mean ± SD)	150.0 ± 17.7	150.0 ± 17.9	149.9 ± 17.5	0.944
DBP, mmHg (mean ± SD)	82.7 ± 11.9	83.6 ± 11.0	81.9 ± 12.5	0.120
Hcy, μmol/L (median, IQR)	10.0 (4.7–12.6)	9.5 (4.0–12.0)	10.9 (8.0–13.0)	0.001
Folate, nmol/l (median, IQR)	5.8 (4.0–8.4)	6.6 (4.1–9.4)	5.2 (3.8–7.8)	0.002
B12, pmol/l (median, IQR)	352.0(253.0–488.0)	355.0 (265.3–495.3)	351.0 (244.5–478.0)	0.500
eGFR, ml/min/1.73 m^2^ (mean ± SD)	92.7 (78.3–104.0)	95.0 (79.8–106.5)	90.1 (77.0–100.8)	0.001
NIHSS score (median, IQR)	1.0 (0.0–3.0)	1.0 (0.0–3.0)	1.5 (1.0–3.0)	0.001
TOAST mechanism				0.008
LAA, *n* (%)	474 (80.1%)	223 (81.1%)	251 (79.2%)	
CE, *n* (%)	58 (9.8%)	17 (6.2%)	41 (12.9%)	
SVO, *n* (%)	40 (6.8%)	21 (7.6%)	19 (6.0%)	
Others, *n* (%)	20 (3.4%)	14 (5.1%)	6 (1.9%)	
HAMD score (median, IQR)	4.0 (2.0–7.0)	4.0 (1.0–7.0)	4.0 (2.0–8.0)	0.016
Depressive symptoms, *n* (%)	139 (23.5%)	60 (21.8%)	79 (24.9%)	0.374

*MMSE, Mini-Mental State Exam; BMI, body mass index; CAD, coronary artery disease; AF, atrial fibrillation; SBP, systolic blood pressure; DBP, diastolic blood pressure; HCY, Homocysteine; eGFR, estimated Glomerular Filtration Rate; NIHSS, National Institute of Health stroke scale; LAA, Large artery atherosclerosis; CE, Cardioaortic embolism; SVO, Small artery occlusion; Others, Other causes; HAMD, Hamilton Depression Scale*.

### Baseline Characteristics of Patients With AIS in Different Hcy Tertiles

Table 2 shows the baseline demographic, clinical, and laboratory characteristics of patients with AIS according to the Hcy tertiles. Levels of Hcy were ≤ 8 μmol/L in the tertile 1 and ≥12 μmol/L in the tertile 3. The incidence of cognitive impairment was significantly higher in the third Hcy tertile than in the first and second Hcy tertiles (29.7 vs. 52.0 and 62.2%, respectively, *p* = 0.008). The median (IQR) scores of the MMSE in the three groups were 25.0 (20.0–28.0), 24.0 (19.0–28.0), and 23.0 (18.0–27), respectively (*p* = 0.005). Patients with higher levels of Hcy had higher BMI, NIHSS scores, a higher percentage of smoking and AF and lower median age, percentage of females, levels of B12, levels of folate, and eGFR.

**Table 2:** Baseline characteristics of patients with AIS in different HCY tertiles.

	**HCY tertiles**			
**Variables**	**Tertile 1**	**Tertile 2**	**Tertile 3**	***P*-value**
	**(*n* = 201)**	**(*n* = 198)**	**(*n* = 193)**	
HCY, μmol/L	≤ 8	8–12	≥12	
MMSE ≤ 24, *n* (%)	94 (46.8%)	103 (52.0%)	120 (62.2%)	0.008
MMSE score (median, IQR)	25.0 (20.0–28.0)	24.0 (19.0–28.0)	23.0 (18.0–27.0)	0.005
Age, year (median, IQR)	63.0 (56.0–69.0)	63.0 (54.0–69.0)	67.0 (59.0–72.0)	0.001
Female, *n* (%)	83 (41.3%)	86 (43.4%)	53 (27.5%)	0.002
BMI (median, IQR)	23.8 (22.6–26.1)	24.2 (22.6–26.6)	23.4 (21.5–26.4)	0.040
Education, year (median, IQR)	4.0 (0.0–6.0)	4.0 (0.0–7.0)	4.0 (0.0–6.0)	0.869
Currently smoking, *n* (%)	92 (46.2%)	80 (40.8%)	102 (53.7%)	0.040
Currently drinking, *n* (%)	72 (36.9%)	66 (34.2%)	68 (37.2%)	0.799
Hypertension, *n* (%)	139 (69.2%)	142 (71.7%)	140 (72.5%)	0.740
Hyperlipidemia, *n* (%)	17 (8.5%)	18 (9.1%)	13 (6.8%)	0.705
Diabetes, *n* (%)	48 (24.0%)	51 (25.8%)	39 (20.5%)	0.467
CAD, *n* (%)	10 (5.1%)	13 (6.6%)	15 (7.9%)	0.515
AF, *n* (%)	8 (4.0%)	16 (8.1%)	23 (11.9%)	0.014
SBP, mmHg (mean ± SD)	149.8 ± 18.0	149.4 ± 17.6	150.8 ± 17.5	0.767
DBP, mmHg (mean ± SD)	81.9 ± 11.1	82.8 ± 12.1	83.4 ± 12.4	0.486
Hcy, μmol/L (median, IQR)	3.8 (3.4–6.2)	10.0 (9.0–11.0)	14.0 (12.1–16.2)	<0.001
Folate, nmol/l (median, IQR)	6.7 (4.6–9.2)	5.4 (3.4–8.2)	5.1 (3.8–8.0)	0.001
B12, pmol/l (median, IQR)	390.0 (265.0–528.0)	366.0(267.0–483.0)	309.0 (223.0–423.0)	0.003
eGFR, ml/min/1.73 m^2^ (mean ± SD)	96.1 (87.0–104.9)	92.8 (80.4–105.3)	84.6 (71.2–99.5)	<0.001
NIHSS score (median, IQR)	1.0 (1.0–2.0)	1.0 (0.0–3.0)	2 (1.0–4.0)	<0.001
TOAST mechanism				0.341
LAA, *n* (%)	161 (80.1%)	160 (80.8%)	153 (79.3%)	
CE, *n* (%)	21 (10.4%)	16 (8.1%)	21 (10.9%)	
SVO, *n* (%)	9 (4.5%)	15 (7.6%)	16 (8.3%)	
Others, *n* (%)	10 (5.0%)	7 (3.5%)	3 (1.6%)	
HAMD score (median, IQR)	3.0 (1.0–7.0)	5.0 (2.0–7.8)	4.0 (2.0–7.0)	0.065
Depressive symptoms, *n* (%)	44 (21.9%)	50 (25.3%)	45 (23.3%)	0.689

*MMSE, Mini-Mental State Exam; BMI, body mass index; CAD, coronary artery disease; AF, atrial fibrillation; SBP, systolic blood pressure; DBP, diastolic blood pressure; HCY, Homocysteine; eGFR, estimated Glomerular Filtration Rate; NIHSS, National Institute of Health stroke scale; LAA, Large artery atherosclerosis; CE, Cardioaortic embolism; SVO, Small artery occlusion; Others, Other causes; HAMD, Hamilton Depression Scale*.

### Association Between Hcy Levels and Cognitive Impairment

Table 3 shows the multivariate logistic analysis for the association between Hcy levels and cognitive impairment. In these regression models, the occurrence of cognitive impairment was considered as a dependent variable and the first Hcy tertile was used as a reference. After adjusting for potential confounders including age, sex, years of education, currently smoking, currently drinking, hypertension, AF, folate, eGFR, NIHSS score, TOAST mechanism, and HAMD score, the third Hcy tertile was independently associated with the prevalence of cognitive impairment [odds ratio (OR) = 2.057, 95% confidence interval (CI) =1.133–3.735, *p* = 0.018]. Moreover, age, years of education, NIHSS score, and cardioaortic embolism (CE) were significantly associated with cognitive impairment in the post-stroke population.

**Table 3:** Multivariate logistic analysis for the association between Hcy levels and cognitive impairment.

	**Univariate analysis**		**Model 1^a^**		**Model 2^b^**	
	**OR (95%CI)**	***p*-value**	**OR (95%CI)**	***p*-value**	**OR (95%CI)**	***p*-value**
**Hcy tertiles**						
**Tertile 1**	**Reference**		**Reference**		**Reference**	
Tertile 2	1.234 (0.833–1.828)	0.294	1.522 (0.928–2.495)	0.096	1.269 (0.720–2.234)	0.410
Tertile 3	1.871 (1.252–2.796)	0.002	2.633 (1.596–4.442)	<0.001	2.057 (1.133–3.735)	0.018
Age, years	1.073 (1.053–1.093)	<0.001	1.055 (1.032–1.079)	<0.001	1.057 (1.027–1.087)	<0.001
Gender, female	3.745 (2.610–5.374)	<0.001	2.224 (1.403–3.525)	0.001	2.092 (0.995–4.398)	0.052
Years of education	1.216 (1.110–1.333)	<0.001	0.720 (0.672–0.772)	<0.001	0.734 (0.677–0.797)	<0.001
Currently smoking	0.419 (0.300–0.585)	<0.001			0.835 (0.435–1.601)	0.587
Currently drinking	0.625 (0.443–0.881)	0.007			1.307 (0.755–2.264)	0.339
Hypertension	1.465 (1.025–2.093)	0.036			1.093 (0.649–1.841)	0.738
AF	1.946 (1.030–3.676)	0.040			0.885 (0.333–2.353)	0.807
Folate	0.514 (0.272–0.971)	0.040			0.978 (0.920–1.041)	0.484
eGFR	0.986 (0.977–0.994)	0.001			0.998 (0.985–1.012)	0.819
NIHSS score	1.142 (1.058–1.233)	0.001			1.160 (1.037–1.298)	0.009
**TOAST mechanism**						
**LAA**	**Reference**				**Reference**	
CE	2.143 (1.184–3.879)	0.012			2.378 (1.008–5.611)	0.048
SVO	0.804 (0.421–1.534)	0.508			1.165 (0.469–2.896)	0.742
Others	0.381 (0.144–1.008)	0.052			1.430 (0.363–5.636)	0.609
HAMD score	1.045 (1.005–1.086)	0.026			0.992 (0.937–1.049)	0.770

*HCY, Homocysteine; AF, atrial fibrillation; eGFR, estimated Glomerular Filtration Rate; NIHSS, National Institute of Health stroke scale; LAA, Large artery atherosclerosis; CE, Cardioaortic embolism; SVO, Small artery occlusion; Others, Other causes; HAMD, Hamilton Depression Scale*.

a *Model 1: adjusted for age, gender and years of education*.

b *Model 2: adjusted for variables in Model 1 plus currently smoking, currently drinking, hypertension, AF, folate, eGFR, NIHSS score, TOAST mechanism, and HAMD score*.

### Baseline Characteristics of Patients With AIS Grouped by Age

Table 4 shows the baseline demographic, clinical, and laboratory characteristics of patients with AIS according to age. Compared with the older group (age ≥ 65 years), the younger age group (age <65 years) had lower levels of Hcy [10.0 (5.0–13.0) vs. 11.0 (4.5–13.0), *p* < 0.001] and lower prevalence of cognitive impairment [126 (41.7%) vs. 191 (65.9%), *p* < 0.001]. The median (IQR) scores of the MMSE in the younger and older groups were 26.0 (21.0–28.0) and 22 (18.0–26.0), respectively (*p* < 0.001). Furthermore, the younger age group had a higher BMI, higher years of education, higher levels of B12, and higher eGFR but had lower DBP. As for the previous history of patients, patients in the older age group were more likely to have a history of hypertension, CAD, and AF. Meanwhile, more cigarette smokers and alcohol drinkers were found in the younger age group.

**Table 4:** Baseline characteristics of patients with AIS grouped by age.

**Variables**	**Total**	**Younger age group (age <65 y)**	**Older age group (age ≥ 65 y)**	***P*-value**
	**(*n* = 592)**	**(*n* = 302)**	**(*n* = 290)**	
Age, year (median, IQR)	64 (57–70)	57 (50–61)	70 (67–74)	<0.001
Female, *n* (%)	222 (37.5%)	106 (35.1%)	116 (40.0%)	0.218
BMI (median, IQR)	23.8 (22.0–26.1)	24.2 (22.4–26.6)	23.1 (21.3–25.5)	<0.001
Education, year (median, IQR)	4.0 (0.0–6.0)	5.0 (0.0–8.0)	3.0 (0.0–6.0)	<0.001
Currently smoking, *n* (%)	274 (46.8%)	157 (53.0%)	117 (40.5%)	0.002
Currently drinking, *n* (%)	206 (36.1%)	119 (40.9%)	87 (31.1%)	0.015
Hypertension, *n* (%)	421 (71.1%)	203 (67.2%)	218 (75.2%)	0.033
Hyperlipidemia, *n* (%)	49 (8.3%)	25 (8.3%)	24 (8.4%)	0.980
Diabetes, *n* (%)	138 (23.5%)	69 (22.9%)	69 (24.0%)	0.749
CAD, *n* (%)	38 (6.5%)	12 (4.0%)	26 (9.1%)	0.013
AF, *n* (%)	47 (7.9%)	12 (4.0%)	35 (12.1%)	0.014
SBP, mmHg (mean ± SD)	150.0 ± 17.7	148.6 ± 18.6	151.4 ± 16.7	0.076
DBP, mmHg (mean ± SD)	82.7 ± 11.9	84.8 ± 12.1	80.5 ± 11.3	<0.001
Hcy, μmol/L (median, IQR)	10.0 (4.7–12.6)	10.0 (5.0–13.0)	11.0 (4.5–13.0)	<0.001
Folate, nmol/l (median, IQR)	5.8 (4.0–8.4)	6.0 (4.2–8.4)	5.2 (3.7–8.5)	0.133
B12, pmol/l (median, IQR)	352.0 (253.0–488.0)	371.0 (258.0–529.0)	337.5 (249.3–445.8)	0.024
eGFR, ml/min/1.73 m^2^ (mean ± SD)	92.7 (78.3–104.0)	100.5 (85.6–109.4)	86.7 (73.1–95.3)	<0.001
NIHSS score (median, IQR)	1.0 (0.0–3.0)	1.0 (0.0–3.0)	1.0 (0.0–3.0)	0.816
TOAST mechanism				0.031
LAA, *n* (%)	474 (80.1%)	243 (80.5%)	231 (79.7%)	
CE, *n* (%)	58 (9.8%)	22 (7.3%)	36 (12.4%)	
SVO, *n* (%)	40 (6.8%)	22 (7.3%)	18 (6.2%)	
Others, *n* (%)	20 (3.4%)	15 (5.0%)	5 (1.7%)	
MMSE ≤ 24, *n* (%)	317 (53.5%)	126 (41.7%)	191 (65.9%)	<0.001
MMSE score (median, IQR)	24.0 (19.0–27.0)	26.0 (21.0–28.0)	22 (18.0–26.0)	<0.001
HAMD score (median, IQR)	4.0 (2.0–7.0)	4.0 (1.0–7.0)	4.0 (2.0–7.0)	0.531
Depressive symptoms, *n* (%)	139 (23.5%)	67 (22.2%)	72 (24.8%)	0.440

*BMI, body mass index; CAD, coronary artery disease; AF, atrial fibrillation; SBP, systolic blood pressure; DBP, diastolic blood pressure; eGFR, estimated Glomerular Filtration Rate; NIHSS, National Institute of Health stroke scale; LAA, Large artery atherosclerosis; CE, Cardioaortic embolism; SVO, Small artery occlusion; Others, Other causes; MMSE, Mini-mental State Examination; HAMD, Hamilton Depression Scale*.

### Multivariate Adjusted OR for the Association Between Hcy Levels and Cognitive Impairment in the Subcategorized Groups of Age

**Figure 1:**
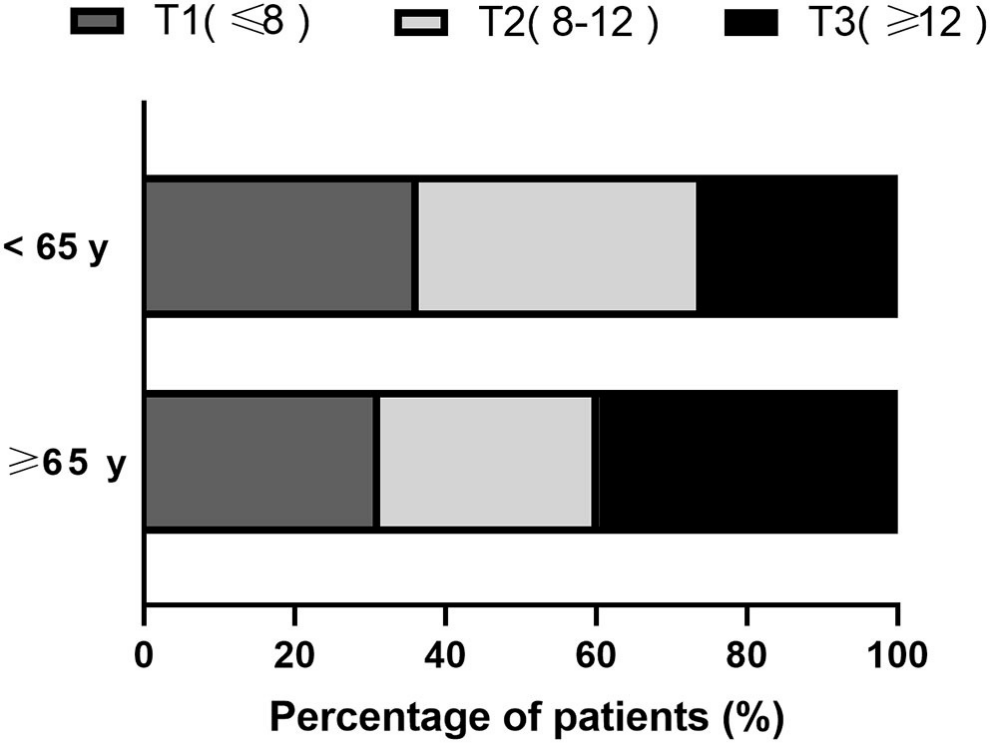
Percentage in the subcategorized groups of age according to Hcy tertiles.

Based on the Hcy tertiles, in the younger age group, 110 (36.4%) had lower Hcy levels and 78 (25.8%) had higher Hcy levels, while in the older age group, 91 (31.4%) had lower Hcy levels and 115 (39.7%) had higher Hcy levels (Figure 1).

Table 5 shows the association between Hcy levels and cognitive impairment in the different age groups. In univariate analyses, the third Hcy tertile was associated with cognitive impairment in the total group with an OR of 1.871 (95% CI = 1.252–2.796, *p* = 0.002) (Table 3). Table 5 shows significant association between the third Hcy tertile and cognitive impairment with a higher risk of the younger age group (OR = 3.156, 95% CI = 1.506–6.613, *p* = 0.002) and the older age group (OR = 1.823, 95% CI = 1.161–3.504, *p* = 0.026). These differences remained significant after adjusting for sex and years of education. After further adjustments for the potential factors detected in the univariate analysis (Model 4: adjusting for sex, years of education, currently smoking, currently drinking, hypertension, AF, Folate, eGFR, NIHSS score, TOAST mechanism, and HAMD score), the association between the third Hcy tertile and cognitive impairment in the younger age group remained significant (Model 4: OR = 3.583, 95% CI = 1.456–8.818, *p* = 0.005). However, the association in the older age group disappeared (Model 4: OR = 1.273, 95% CI = 0.547–2.961, *p* = 0.576). Notably, in the younger age group, patients in the second Hcy tertile group were also associated with an increased risk of cognitive impairment with an OR of 2.625 (95% CI = 1.351–5.102, *p* = 0.004) in univariate analyses. These differences remained significant after adjusting for confounding factors and risk factors (Model 4: OR = 2.266, 95% CI = 1.042–4.926, *p* = 0.039).

**Table 5:** Multivariate adjusted odds ratios for the association between Hcy levels and cognitive impairment in the subcategorized groups of age.

	**Univariate analysis**		**Model 3^a^**		**Model 4^b^**	
	**OR (95%CI)**	***p*-value**	**OR (95%CI)**	***p*-value**	**OR (95%CI)**	***p*-value**
**Younger age group (age** **<** **65 y)**						
**Hcy tertiles**						
**Tertile 1**	**Reference**		**Reference**		**Reference**	
Tertile 2	2.625 (1.351–5.102)	0.004	2.199 (1.120–4.317)	0.022	2.266 (1.042–4.926)	0.039
Tertile 3	3.156 (1.506–6.613)	0.002	3.767 (1.770–8.017)	0.001	3.583 (1.456–8.818)	0.005
**Older age group (age** **≥** **65 y)**						
**Hcy tertiles**						
**Tertile 1**	**Reference**		**Reference**		**Reference**	
Tertile 2	1.291 (0.701–2.376)	0.413	0.941 (0.439–2.017)	0.875	0.661 (0.273–1.600)	0.358
Tertile 3	1.823 (1.161–3.504)	0.026	2.147 (1.035–4.456)	0.040	1.273 (0.547–2.961)	0.576

a *Model 3: adjusted for gender and years of education*.

b *Model 4: adjusted for variables in Model 1 plus currently smoking, currently drinking, hypertension, AF, folate, eGFR, NIHSS score, TOAST mechanism, and HAMD score*.

## Discussion

To our knowledge, this is the first study to discuss and analyze the relationship between Hcy levels, age, and cognitive impairment in a post-stroke population. Our study demonstrated that elevated Hcy levels in the acute phase of ischemic stroke were independently associated with cognitive impairment in the post-stroke population. In the comparison of different age groups (≥65 and <65 years), we found that elevated Hcy levels played a stronger role in the younger age group than in the older age group. After adjusting for the confounding factors, the association remained significant in the total group and the younger age group but disappeared in the older age group. The results indicated the age-dependent effect of Hcy on cognitive impairment in the post-stroke population. Of note, moderately raised serum Hcy (≥12 μmol/L in the total group and >8 μmol/L in the younger age group) increased the risk of 1-month cognitive impairment in the post-stroke population, as reflected by the MMSE scores.

In addition to age and years of education, the conventional factors of PSCI, we also found that higher NIHSS scores were independently associated with cognitive impairment in the post-stroke population, which has been proven in previous studies (34, 35). We also found that CE was associated with a higher prevalence of cognitive impairment in stroke survivors independently. In a previous study, PSCI was found to be common for all stroke subtypes (36), which was inconsistent with this study. The insufficient sample size of the CE might have imposed some biases in the results. In our study, large artery atherosclerosis accounted for a large proportion (80.1%), while CE accounted for only 9.8%. Therefore, further studies are needed to explore the relationship between the TOAST subtypes and PSCI. Moreover, fewer cigarette smokers were found in patients with cognitive impairment and moderately elevated Hcy levels (T2). However, previous studies showed that smoking was a risk factor for PSCI (4) and high Hcy levels (17, 18). The contrary results in this study may be attributed to the lower proportion of males among these patients.

This study found an independent association between Hcy levels and cognitive impairment in the post-stroke population. However, the results were contradictory, as mentioned earlier. One study conducted in 169 patients with stroke demonstrated that the linear relationship between Hcy levels and acute MMSE scores disappeared after adjusting for stroke subtypes (15). Another study failed to find a relationship between the plasma Hcy levels and the 27-month cognitive changes after stroke in 170 well-recovered elderly patients (16). The reasons for the contradictory results of previous studies remain unclear. One possible explanation for this discrepancy is age. The risk for AD increases rapidly with age, doubling every 5 years after the age of 65 (23). Similarly, a review of PSD revealed that age ≥ 65 years was a risk factor for PSD (4).To determine whether there is an age-dependent association between Hcy and cognitive impairment in stroke survivors, we divided the total group into two subgroups, ≥65 and <65 years.

As mentioned earlier, an independent association has been reported between Hcy and PSCI with a variable time of outcome assessments from 2 months and up to 3 years after stroke (12–14). We observed an age difference in the relationship between Hcy levels and cognitive impairment in the post-stroke population. None of the aforementioned studies performed an age-specific analysis. Accumulating evidence indicated a relationship between Hcy levels, age, and stroke (19–21). For example, a prospective study revealed a strong, graded, and significant association between Hcy and stroke in young Asian patients with ischemic stroke (21). Moreover, Wang et al. (37) found no association between Hcy and functional outcome after stroke among elderly patients. In addition, a study based on cognitively healthy subjects discovered an age-dependent association between Hcy and cognitive decline and demonstrated that younger patients had a stronger association (22). Our findings regarding serum Hcy levels and cognitive impairment in the post-stroke population support and extend the previous studies. Indeed, we found that elevated Hcy could be an independent risk factor for cognitive impairment only in younger patients but not in older patients. Thus, the association between Hcy and cognitive impairment in the post-stroke population was age-dependent, suggesting that it could be useful in screening and targeting younger patients who need to lower their Hcy levels to improve the cognitive prognosis of ischemic stroke.

In our study, we also found that moderately raised Hcy (≥12 μmol/L in the total group and >8 μmol/L in the younger age group) increased the risk of cognitive impairment in the post-stroke population, which has not been studied yet. Previous studies only considered Hcy as a continuous variable in analysis and did not explore the graded association between Hcy and PSCI (12–14). The reference values for plasma Hcy ranged from 5 to 15 μmol/L. The latest guideline for the prevention and treatment of hypertension in China in 2010 has regarded an increase of Hcy ≥ 10 μmol/L as one of the important risk factors for cardiovascular and cerebrovascular diseases. Moreover, an International Consensus Statement in 2018 indicated that moderately elevated plasma total Hcy (>11 μmol/L) was one of the causes of age-related cognitive decline and dementia (38). Notably, we found a lower range of Hcy concentrations (>8 μmol/L), which would significantly increase the prevalence of cognitive impairment in younger stroke survivors. Therefore, Hcy levels in patients with stroke should be well-monitored, especially in younger patients. In other words, Hcy-lowering treatment should be administered once Hcy concentrations reach over 8 μmol/L in younger patients with stroke. Only one study has explored the association between Hcy-lowering therapies and cognitive impairment after stroke. Nevertheless, Hankey et al. (39) failed to discover an association between daily Hcy-lowering treatment and cognitive impairment in patients with previous stroke or transient ischemic attack. But in the aforementioned study, the Hcy-lowering treatment was administered without adjusting for age and Hcy levels. Interestingly, a *post-hoc* analysis from a clinical trial showed that younger patients may benefit the most from Hcy-lowering therapies (40). Despite the negative results in clinical studies using Hcy-lowering therapies to improve cognitive performance after stroke, the age-dependent effect on the association between Hcy and cognitive impairment in the post-stroke population is encouraging to pursue a feasible and effective treatment option in a specific subgroup of patients divided by age.

Homocysteine is postulated to cause PSCI *via* various mechanisms. Elevated Hcy is well-known to exert cytotoxic and pro-inflammatory effects, leading to vascular endothelial dysfunction and lipid metabolic disorders that cause thrombosis and arteriosclerosis (8–10, 41). All of these result in cerebrovascular disease, which indirectly causes vascular cognitive impairment (VCI). Moreover, Hcy neurotoxicity involves an increase in glutamate excitotoxicity (42), accelerating the development of oxidative stress in the hippocampal neurons (43), amyloid and tau protein accumulation (11), apoptosis (44), and neuronal death (45) contributing to the pathogenesis of AD. In short, elevated Hcy involves not only in VCI but also in the pathogenesis of AD, which has also been observed in the mechanisms of PSCI (1). However, these cannot explain why the association between Hcy and cognitive impairment exists only in younger patients. One possible explanation for this finding was that the association between Hcy levels and cognitive impairment in the post-stroke population was not causal among elderly patients. In other words, the harmful effects of elevated Hcy could be masked by other vascular risk factors, such as hypertension, diabetes, and AF. The prevalence rates of hypertension and diabetes were 75.2 and 24.0% in the older age group, respectively, which were much higher than the rate of the younger age group in the present study and the rates reported in other studies.

This study has certain limitations. First, this was a single-center and prospective study with a lack of baseline MMSE score. Therefore, we could not establish causality between Hcy and cognitive impairment in the post-stroke population. First, multicenter studies are needed to confirm these results and verify the justification of the Hcy tertiles. Second, cognitive function was merely measured by MMSE, other stroke-specific measures and more detailed neuropsychological assessments should be applied together with MMSE to evaluate the cognitive function accurately in the future study. Third, longer follow-up periods are needed to explore the effect of elevated Hcy on cognitive impairment in the post-stroke population. Fourth, we did not consider radiological parameters in the analysis. Fifth, the medications of the patients were not recorded on admission and during the follow-up, which may cause some bias in this study. Finally, patients with aphasia or other serious conditions, and those aged > 80 years, were not included in our study. Meanwhile, patients enrolled in our sample analysis tended to have lower NIHSS scores and mostly minor strokes on admission. These may have underestimated the actual incidence of cognitive impairment after stroke and diminished the representativeness of the cohort, thus limiting the generalization of the results. Larger sample size and prolonged follow-up should be taken into account in the future.

## Conclusion

In summary, our study suggested a graded association between elevated Hcy and cognitive impairment in the post-stroke population. We found that the association was stronger in younger patients but not in older patients. Therefore, Hcy levels in patients with stroke should be well-monitored, especially in younger patients. Further studies should be conducted among participants with different social and cultural backgrounds to replicate our findings.

## Data Availability Statement

The original contributions presented in the study are included in the article/supplementary material, further inquiries can be directed to the corresponding authors.

## Ethics Statement

The studies involving human participants were reviewed and approved by Ethics Committee of the First Affiliated Hospital of Wenzhou Medical University. The patients/participants provided their written informed consent to participate in this study.

## Author Contributions

J-CH and S-NZ designed the study. S-NZ, J-HC, and LC wrote the manuscript. S-NZ did the statistical analyses. J-HC, LC, K-LF, W-WR, M-JX, Y-BC, D-DG, H-RC, X-QL, J-YS, and G-QL screened and extracted data. J-CH and G-QH supervised the study. All the authors have made an intellectual contribution to the manuscript and approved the submission.

## Conflict of Interest

The authors declare that the research was conducted in the absence of any commercial or financial relationships that could be construed as a potential conflict of interest.
