# Corrigendum: Age-Dependent Association Between Elevated Homocysteine and Cognitive Impairment in a Post-stroke Population: A Prospective Study

**DOI:** 10.3389/fnut.2021.736283

**Published:** 2021-09-07

**Authors:** Shengnan Zhou, Jiahao Chen, Lin Cheng, Kaili Fan, Minjie Xu, Wenwei Ren, Yunbin Chen, Dandan Geng, Haoran Cheng, Xiaoqian Luan, Jiaying Song, Gangqiang Lin, Guiqian Huang, Jincai He

**Affiliations:** ^1^Department of Neurology, The First Affiliated Hospital of Wenzhou Medical University, Wenzhou, China; ^2^School of Mental Health, Wenzhou Medical University, Wenzhou, China

**Keywords:** homocysteine, age, ischemic stroke, cognitive impairment, Mini-Mental State Exam

In the published article, there was an error regarding the affiliation for Minjie Xu. As well as having affiliation **1**, she should also have affiliation **2**. The corrected affiliation was Minjie Xu^1,2^.

The authors apologize for these errors and state that this does not change the scientific conclusions of the article in any way. The original article has been updated.

## Publisher's Note

All claims expressed in this article are solely those of the authors and do not necessarily represent those of their affiliated organizations, or those of the publisher, the editors and the reviewers. Any product that may be evaluated in this article, or claim that may be made by its manufacturer, is not guaranteed or endorsed by the publisher.

